# Combined Application of Gadoxetic Acid Disodium-Enhanced Magnetic Resonance Imaging (MRI) and Diffusion-Weighted Imaging (DWI) in the Diagnosis of Chronic Liver Disease-Induced Hepatocellular Carcinoma: A Meta-Analysis

**DOI:** 10.1371/journal.pone.0144247

**Published:** 2015-12-02

**Authors:** Xiang Li, Chenxia Li, Rong Wang, Juan Ren, Jian Yang, Yuelang Zhang

**Affiliations:** 1 Department of Diagnostic Radiology, The First Affiliated Hospital of Xi’an Jiaotong University, Xi’an, Shaanxi Province, China; 2 Department of Radiotherapy, The First Affiliated Hospital of Xi’an Jiaotong University, Xi’an, Shaanxi, China; Affiliated Hospital of North Sichuan Medical College, CHINA

## Abstract

**Objective:**

Gadoxetic acid disodium (Gd-EOB-DTPA) is a magnetic resonance imaging (MRI) contrast agent to target the liver cells with normal function. In clinical practice, the Gd-EOB-DTPA produces high quality hepatocyte specific image 20 minutes after intravenous injection, so DWI sequence is often performed after the conventional dynamic scanning. However, there are still some disputes about whether DWI sequence will provide more effective diagnostic information in clinical practice. This study aimed to explore the diagnostic value of combining Gd-EOB-DTPA-enhanced MRI and DWI in the detection of hepatocellular carcinoma (HCC) in patients with chronic liver disease.

**Methods:**

A systematic literature search was performed in the PubMed and Cochrane library database up to March 2015. The quality assessment of diagnostic accuracy studies (QUADAS) was used to evaluate the quality of studies. Heterogeneous test on the included literature was performed by using the software Review Manager 5.3. The MetaDiSc 1.4 software was used to calculate the pooled sensitivity, specificity, positive likelihood ratio and negative likelihood ratio; meanwhile the summary receiver operating characteristics (SROC) curve was drawn to compare the diagnostic performance.

**Results:**

A total of 13 literatures were included in this study. In 8 literatures regarding HCC diagnosis based on Gd-EOB-DTPA-enhanced MRI, the pooled sensitivity: 0.90 (95% confidence interval (CI): 0.88–0.93); specificity: 0.89 (95% CI: 0.85–0.92); positive likelihood ratio: 8.60 (95% CI: 6.20–11.92); negative likelihood ratio: 0.10 (95% CI: 0.08–0.14) were obtained. The area under curve (AUC) and Q values were 0.96 and 0.90, respectively. In 5 literatures relating to HCC diagnosis by combination of Gd-EOB-DTPA-enhanced MRI and DWI sequence, the pooled sensitivity: 0.88 (95% CI: 0.85–0.91), specificity: 0.96 (0.94–0.97), positive likelihood ratio: 19.63 (12.77–30.16), negative likelihood ratio: 0.10 (0.07–0.14) were obtained. The AUC value was 0.9833 and Q value was 0.9436. The AUC value of comprehensive evaluation method was significantly higher than that of Gd-EOB-DTPA-enhanced MRI alone(*P*<0.05).

**Conclusion:**

Combination of Gd-EOB-DTPA-enhanced MRI and DWI sequence significantly improves in both the diagnostic accuracy and specificity of chronic liver disease-associated HCC.

## Introduction

As a most common malignant tumors, hepatocellular carcinoma (HCC)’s mortality ranks third in all death of malignant tumors in developing countries [1]. Chronic liver disease including cirrhosis, is one of the most important factors contributing to the multistep progression of hepatocarcinogenesis, from benign regenerative nodules to early HCC, and finally to overt HCC [2].

Currently the diagnosis of hepatocellular carcinoma (HCC) is primarily based on imaging. Gadoxetic acid disodium (Gd-EOB-DTPA) is a liver specific contrast agent with unique EOB group, which can be uptake specifically by normal hepatocyte (about 50% uptake rate), thereby producing enhancement effect in liver cells 20 min after Gd-EOB-DTPA administration [3]. Therefore, it could provide useful information to distinguish abnormal hepatocytes (including HCC) from normal ones [4]. Golfieri. R. et al had shown that EOB-MRI has the capability of identifying the HCC precursors and portraying their biological behaviors, thus rapidly becoming a key imaging tool for the diagnosis of HCC and its precursors [5]. Furthermore, one study demonstrated that magnetic resonance imaging (MRI) using gadoxetic acid provided more accurate diagnosis in discriminating focal nodular hyperplasia (FNH) from hepatic adenoma (HA), identification of early HCC and pre-operative assessment of metastasis in liver parenchyma [6]. Diffusion-weighted imaging (DWI) can provide cellular information of HCC and also has been widely applied in lesion detection, lesion characterization, and assessment of treatment response to chemotherapeutic agents. Some researchers had revealed that diffusion-weighted MRI can provide additional information to differentiate HCC from DN or other pseudotumoral lesions [7,8]. Actually, diffusion-weighted MRI does provide enhanced diagnostic value in the detection and characterization of focal liver lesions [9,10]. Recently, Chen. J. et al had demonstrated that DWI had excellent and moderately high diagnostic accuracy for the detection of well-differentiated HCC and poorly-differentiated HCC, respectively [11].

In clinical practice, the Gd-EOB-DTPA produces high quality hepatocyte specific image 20 minutes after intravenous injection, so DWI sequence is often performed after the conventional dynamic scanning for both shorten the duration of the examination as a whole and provide target cellular and architectural changes through the differences in tissue diffusivity. Saito K. et al research had shown that the injection of Gd-EOB-DTPA followed by scan of DWI sequence did not affect the enhanced HCC diagnosis by Gd-EOB-DTPA [12]. There are still some disputes about whether DWI sequence will provide more effective diagnostic information in clinical practice. Some studies have shown that combined DWI sequences and hepatocyte specific contrast agent is conducive to differentiation of benign and malignant hepatic lesions [13–15]. However, some recent reports suggested that the added DWI sequence cannot significantly improve detection efficiency of malignant liver disease [16–18]. In this study, we performed meta-analysis in view of evidence based medicine, to make a comprehensive, objective and accurate evaluation on the HCC detection efficiency by combined application of Gd-EOB-DTPA-enhanced MRI and DWI sequences.

## Materials and Methods

### Literature Search

Literatures were searched from PubMed, Cochrane library database and reference as the main source of data, using terms combining any two keywords from ("diffusion-weighted magnetic resonance imaging" or "DWMRI" or "DWI"), ("Gadoxetic-acid-enhanced MRI" or "Gd-EOB-DTPA enhanced MRI") and ("hepatocelluar carcinoma" or "liver neoplasms")(Last search update to March 15, 2015). Literatures were screened from the title, abstract, intensive reading full-text, and reviews, comments, letters, animal models and case reports were excluded.

### Inclusion and Exclusion Criteria

Inclusion criteria: (1) English literature; (2) subjects were patients with chronic liver disease with more than 30 cases; (3) the objective of research was to evaluate HCC diagnostic efficiency in hepatocyte specific phase or combined application of hepatocyte specific phase and DWI; (4) gold standard for HCC diagnosis was pathological examination or imaging follow-up; (5) data analysis was based on the number of lesions, the original data can be directly or indirectly provided, and indicators for HCC diagnosis can be calculated, including the true positive (TP) value, false (FP) positive, false negative (FN) value, true negative (TN) value; (6) the quality of literature was evaluated using the quality assessment of diagnostic accuracy studies (QUADAS), and when there were more than 9 "Yes" from a total of 14 questions, the literature can be incorporated into the study, when there were more than 4 "No" or "Unclear", the literature will be excluded [19,20]. Exclusion criteria: (1) review literature, system evaluation, letters, comments or animal models; (2) in addition to HCC, the study object was also suffering from other malignant lesions; (3) the research subject was less than 30 cases.

### Data Extraction and Quality Evaluation

Data extraction was performed by three investigators independently who also performed the database searches, and any lack of clarity or disagreement was resolved through discussion. The investigators abstracted data from each study to obtain information on author, publication year, sample size, number of lesions, characteristics of the study population (age, gender), the size of the hepatocellular carcinoma, gold standard selection, types of liver disease and study design type (prospective, retrospective), diagnostic method, equipment and directly or indirectly obtained indicators really positive (TP) value, false positive (FP) value, false negative (FN) value, true negative (TN) value, sensitivity (Se) and specificity (Sp). The quality of relevant studies were further evaluated using the quality assessment of diagnostic accuracy studies (QUADAS) tool. It includes 14 items to assess risk of bias, source of variation and reporting quality. The answer to each item was “yes,” “no,” or “unclear”.

### Statistical Analysis

Review Manager 5.3 software was used to analyze the heterogeneity of the research, and the Q test was applied to calculate the inconsistency index I^2^ value. Due to the low sensitivity of Cochrane Q test, the significance level α = 0.1 was adopted for conservation [21,22], and P > 0.1 indicates there is no statistical heterogeneity between studies, P < 0.1 indicates there is heterogeneity. I^2^ was used to quantitatively evaluate heterogeneity, and when I^2^ < 25%, fixed effect model was used for meta-analysis; when 25% < I^2^ < 50%, random effect model was used; when I^2^ > 50%, the sources of heterogeneity was analyzed firstly, if there was no obvious clinical heterogeneity and the sources of heterogeneity cannot be found, the random effect model was used. The MetaDiSc 1.4 software was used to perform meta-analysis based on various indicators, including sensitivity (Se), specificity (Sp), positive likelihood ratio (+LR), negative likelihood ratio (−LR). All indicators were represented as pooled results and the 95% confidence interval (CI). In addition, the summary receiver operating characteristics (SROC) curve was constructed to make comprehensive evaluation on the diagnostic tests. Z tests was performed to determine the difference in AUC between the two diagnostic methods, with P<0.05 regarded as statistically significant. In this paper, funnel plot was drawn to judge whether there is a publication bias. If there is no bias, the graph is symmetric funnel shape. On the contrary, any deviations in the graph prove the existence of publication bias. However, there is no need for publication bias test for the diagnostic method from less literature.

## Results

### Literature Selection

**Fig 1:**
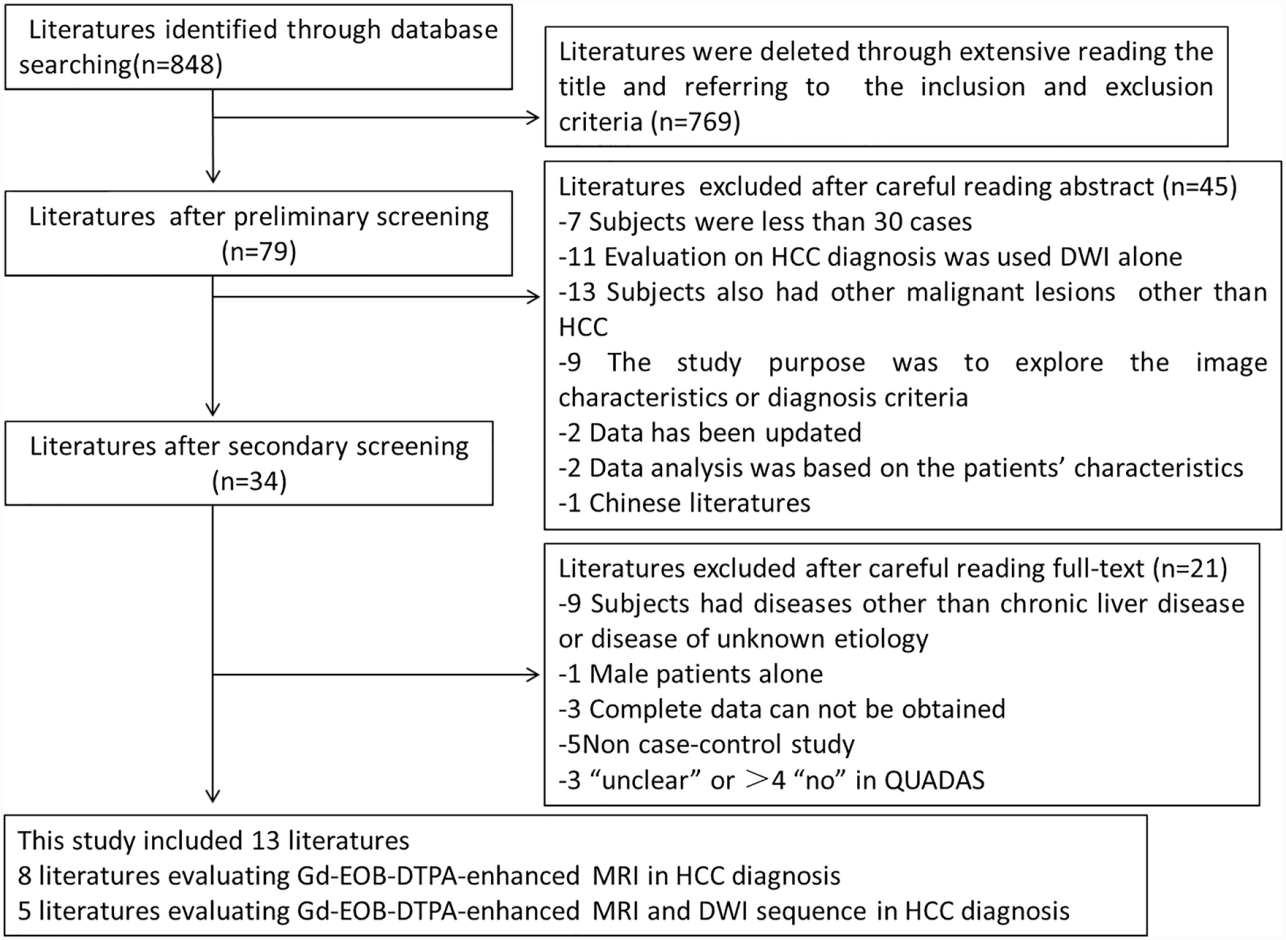
Flow diagram of study selection.

Database was searched using keywords and the search scope was expanded by reference literature, and a total of 848 articles were obtained. Firstly, through extensive reading the title and abstract, a total of 769 literatures were excluded, including reviews, comments, letters and animal models. After careful reading abstract, 45 articles were excluded. Then after intensive reading the full-text, other 21 articles were excluded. Finally this study included 13 articles, including 8 articles regarding HCC diagnosis based on Gd-EOB-DTPA-enhanced MRI [23–30], and 5 articles relating to HCC diagnosis by combination of Gd-EOB-DTPA-enhanced MRI and DWI sequence [31–35]. The flow chart of the literature selection is shown in Fig 1.

### Data Extraction and Quality Assessment

This study included 13 literatures, with the case number of 945 (485 cases of evaluation on Gd-EOB-DTPA-enhanced MRI, 460 cases of evaluation on combined application of Gd-EOB-DTPA-enhanced MRI and DWI), and total lesion number of 1385 (720 lesions of evaluation on Gd-EOB-DTPA-enhanced MRI, 665 lesions of evaluation on combined application of Gd-EOB-DTPA-enhanced MRI and DWI). Detailed information of the included literature is shown in Table 1. In addition, the various indicators obtained directly or indirectly from each study were shown in Table 2, including true positive (TP) value, false positive (FP) value, false negative (FN) value, true negative (TN) value, sensitivity (Se) and specificity (Sp). Among 8 literatures of evaluation on Gd-EOB-DTPA-enhanced MRI, there are 2 literatures with lesion size ≤ 3.0 cm [27,29], 2 literatures used pathological diagnosis as the only gold standard [28,29], and 2 literatures were prospective study [26,28]. Among 5 literatures of evaluation on combined application of Gd-EOB-DTPA-enhanced MRI and DWI, there was 1 literature with lesion size ≤ 3.0 cm [34], and all 5 literatures used pathological diagnosis as the only gold standard. In literature of Lee MH et al [32], only part of the patients underwent DWI, and detail characteristics (age, gender) and lesion size had not been record. Additionally, of the 5 literatures, 2 literatures [32,35] with b values of 0 and 800 sec/mm^2^, 2 literatures [31,34] with 0, 100 and 800 sec/mm^2^, and 1 literature [33] with 1000 sec/mm^2^. Hyperintense on DWI was considered as primary assessment criteria in sensitivity and specificity analysis for hepatocellular carcinoma, although apparent diffusion coefficient which is used to other analysis was calculated in one literature [35]. One study [8] found DWI with b value of 1000 sec/mm^2^ has a sensitivity of 79.0% according to the difference of signal intensity between liver lesions and adjacent hepatic parenchyma. Another research [34] demonstrated on b = 800 sec/mm^2^ images the sensitivity performed by different observers is 79.9%, 77.7% and 78.8% respectively, which is similar to b = 1000 sec/mm^2^ images. So, various b values have a little influence on the result of this research. The evaluations on the design characteristics based on the QUADAS tool are shown in Table 3.

**Table 1:** Basic characteristics of included literatures.

Author	Year	Cases	Lesion	M/F	Age(Scope)	Size(Scope)	Gold standard	Study design	Basic disease	Diagnosis	Equipment
**Ahn SS**[23]	2010	59	113	50/9	57 (29–75)	2.8 (0.4–11)	P^c^/I^d^	R^e^	C^g^	1^j^	1.5T+3.0T
**Baek CK**[24]	2012	51	73	43/8	ND^a^ (32–80)	2.98(0.2–10)	P/I	R	C/H^h^	1	3.0T
**Bashir MR**[25]	2013	100	125	57/43	57.9(29–91)	ND	P/I	R	C	1	1.5T+3.0T
**Di Martino M**[26]	2010	58	109	39/19	63(35–84)	1.8(0.3–7.0)	P/I	P^f^	C	1	1.5T
**Haradome H**[27]	2011	52	60	60/15	54.7 (42–67)	1.74 (0.5–2.8)	P/I	R	C	1	1.5T
**Kim SH**[28]	2009	62	83	54/8	55(31–76)	2.9 (0.5–10.5)	P	P	C/H	1	3.0T
**Rhee H**[29]	2012	34	60	30/4	57(30–66)	1.44 (0.4–3.0)	P	R	C/H	1	3.0T
**Sun HY**[30]	2010	69	97	56/13	ND	1.37 ± 0.41	P/I	R	C	1	3.0T
**Hwang J**[31]	2014	63	160	54|9	52(33–68)	2(0.5–7.8)	P	R	C/H	2^k^	3.0T
**Lee MH**[32]	2011	40	42	UN	UN^b^	UN	P	R	C	2	3.0T
**Ooka Y**[33]	2013	54	87	40/14	68.8±10.5	1.84(0.3–6.5)	P	R	C	2	1.5T
**Park MJ**[34]	2012	260	323	185/75	55.1±7.9	• ≤2.0	P	R	L^i^	2	3.0T
**Inchingolo R**[35]	2015	43	53	34/9	66(46–82)	2.17(1–4)	P	R	C	2	1.5T

^a^ND, not documented.

^b^UN, unclear.

^c^P, Pathological follow up.

^d^I, Imaging follow up.

^e^R, Retrospective study.

^f^P, Prospective study.

^g^C, Cirrhosis.

^h^H, Hepatitis.

^i^L, Chronic liver disease.

^j^1, Gd-EOB-DTPA-enhanced MRI

^k^2, Gd-EOB-DTPA-enhanced MRI and DWI

**Table 2:** Various indicators of included literatures.

Author	Year	TP^a^	FP^b^	FN^c^	TN^d^	Se^e^	Sp^f^
**Ahn SS**[23]	2010	77	2	7	27	91.7	93.1
**Baek CK**[24]	2012	67	4	6	33	91.8	89.2
**Bashir MR**[25]	2013	63	14	7	42	90.9	74.2
**Di Martino M**[26]	2010	74	2	13	20	85	91
**Haradome H**[27]	2011	52	4	8	35	86.7	89.7
**Kim SH**[28]	2009	78	1	5	48	94	97.96
**Rhee H**[29]	2012	27	5	2	26	93.1	83.9
**Sun HY**[30]	2010	41	2	3	51	96.2	93.2
**Hwang J**[31]	2014	89	3	24	44	78.8	91.5
**Lee MH**[32]	2011	21	2	10	8	68	73
**Ooka Y**[33]	2013	83	15	4	339	95.4	95.7
**Park MJ**[34]	2012	165	4	14	140	92.4	97.5
**Inchingolo R**[35]	2015	41	0	1	11	97.6	100

^a^TP, true positive value.

^b^FP, false positive value.

^c^FN, false negative value.

^d^TN, true negative value.

^e^Se, sensitivity.

^f^Sp, specificity.

**Table 3:** Evaluation on included and excluded literatures. ^a^

Author	Case representation	Clear inclusion criteria	Gold standard reliability	Time between gold standard and test	Sample accepts criteria	Same gold standard	Gold standard independence	Clear test description	Clear gold standard description	Blinded test	Blinded gold standard	Obtained same clinical data	Difficult interpretation of results	Exit explanation
**Included literature**														
**Ahn SS**	Y	Y	Y	Un	Y	N	Y	Y	Y	Y	Un	Y	Y	Y
**Baek CK**	Y	Y	Y	Un	Y	N	Y	Y	Y	Y	Un	Y	Y	Y
**Bashir MR**	Y	Y	Y	Y	Y	N	Y	Y	Y	Un	Un	Y	Y	Y
**Di MartiN M**	Y	Y	Y	Y	Y	N	Y	Y	Y	Y	Y	Y	Y	Y
**Haradome H**	Y	Y	Y	Un	Y	Y	Y	Y	Y	Y	Un	Y	Y	Y
**Kim SH**	Y	Y	Y	Y	Y	Y	Y	Y	Y	Y	Y	Y	Y	N
**Rhee H**	Y	Y	Y	Un	Y	Y	Y	Y	Y	Y	Un	Y	Y	Y
**Sun HY**	Y	Y	Y	Y	Y	Y	Y	Y	Y	Y	Un	Y	Y	Y
**Inchingolo R**	Y	Y	Y	Y	Y	Y	Y	Y	Y	Y	Un	Un	Y	N
**Hwang J**	Y	Y	Y	Un	Y	Y	Y	Y	Y	Y	Un	Y	Y	Y
**Lee MH**	Y	Y	Y	Y	Y	Y	Y	Y	Y	Y	Un	Y	Y	N
**Ooka Y**	Y	Y	Y	Un	Y	N	Y	Y	Y	Y	Un	Y	Y	Y
**Park MJ**	Y	Y	Y	Y	Y	Y	Y	Y	Y	Y	Un	Y	Y	Y
**Excluded literature** ^b^														
**Baird AJ**[36]	Y	Un	Y	Y	Y	Y	Y	Y	N	Y	Un	Un	Y	N
**Granito A**[37]	Y	Y	Y	Un	Y	N	Un	Y	N	Y	Un	Y	Y	Y
**Takahashi M**[38]	N	Y	Y	Y	Y	Y	Y	Y	N	Un	Un	N	Y	Y

^a^The quality of literature was evaluated using the quality assessment of diagnostic accuracy studies (QUADAS) [20]

^b^literature excluded by more than 4 in "no" or "Unclear" answer in QUADAS

Y, Yes; Un, Unclear; N, No.

### Heterogeneity Test and Publication Bias

**Fig 2:**
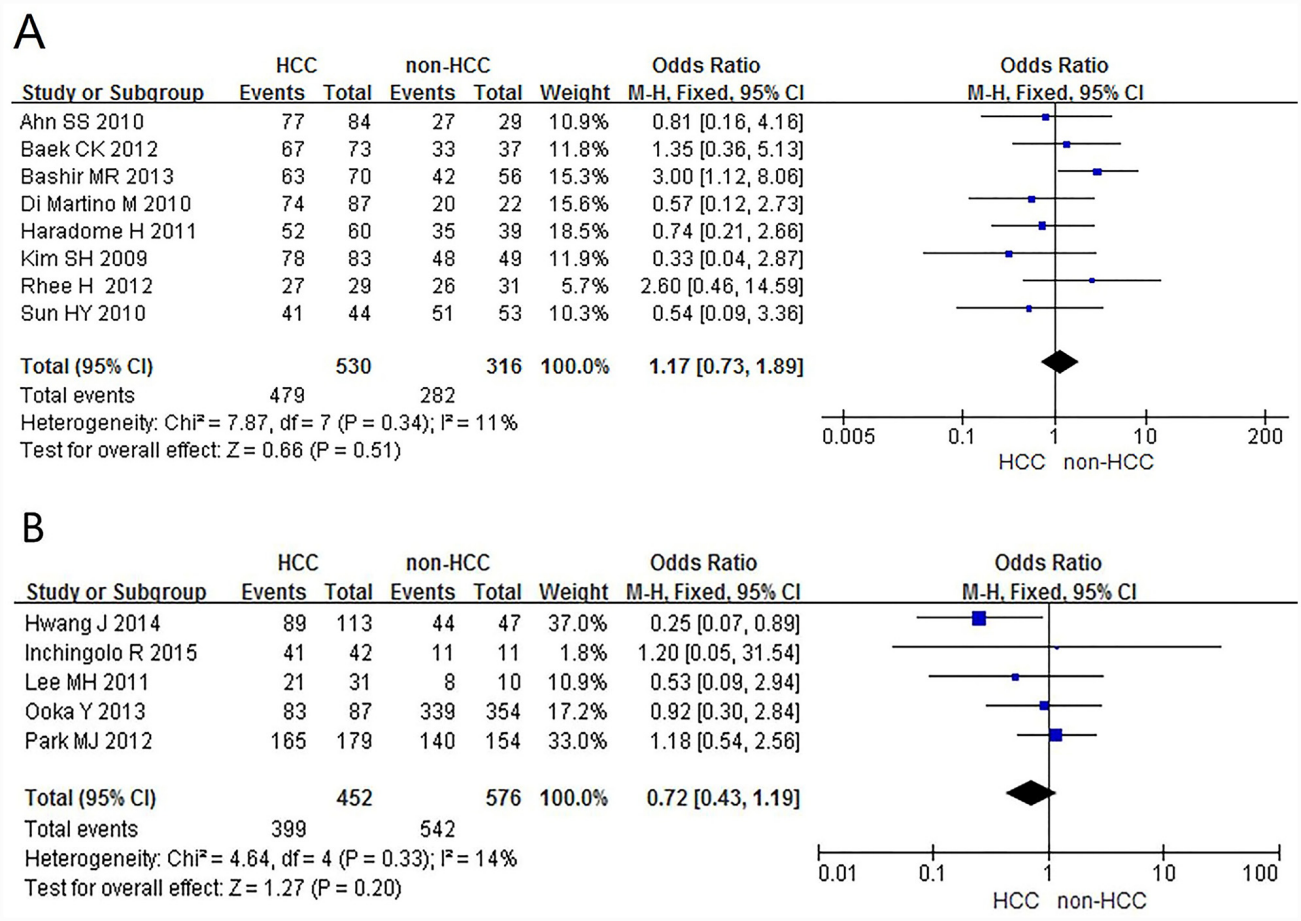
Heterogeneity test results. (A) the estimates for Gd-EOB-DTPA-enhanced MRI in HCC diagnosis. (B) the estimates for combination of Gd-EOB-DTPA-enhanced MRI and DWI sequence in HCC diagnosis.

**Fig 3:**
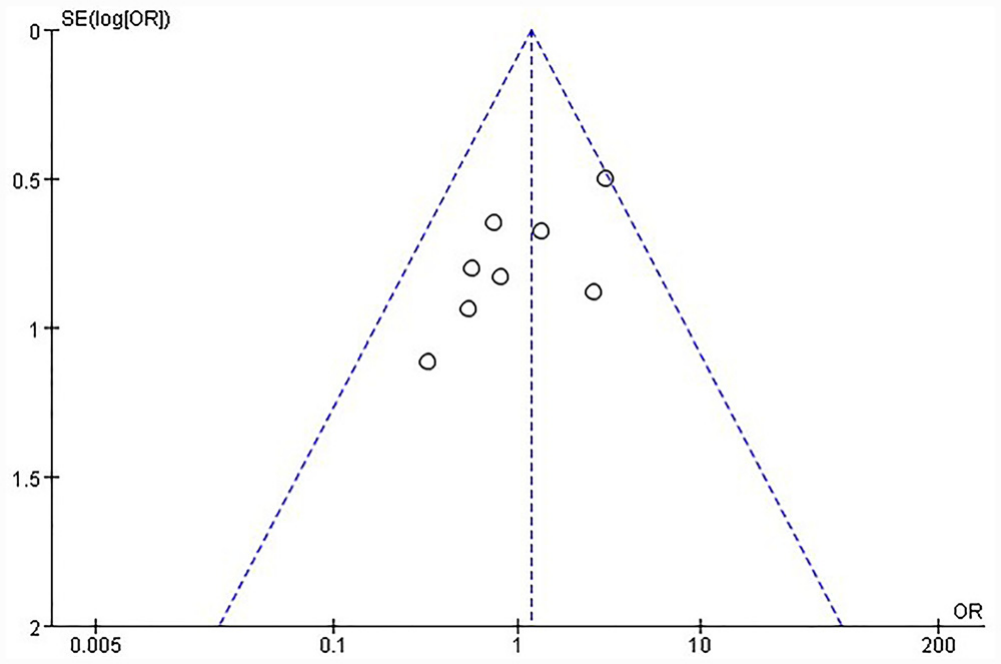
Funnel plot of the estimates for Gd-EOB-DTPA-enhanced MRI in HCC diagnosis. (A) sensitivity analysis. (B) specificity analysis. (C) positive likelihood ratio analysis. (D) negative likelihood ratio analysis.

Fig 2A shows heterogeneity test results from included literatures regarding HCC diagnosis based on Gd-EOB-DTPA-enhanced MRI. Clearly, there is no statistical heterogeneity between different studies (*P* = 0.34), and the fixed effect model can be used for meta-analysis (I^2^ = 11%). The summary diagnostic OR was 1.17 (95% CI: 0.73–1.89); Fig 2B displays heterogeneity test results from included literatures relating to HCC diagnosis by combination of Gd-EOB-DTPA-enhanced MRI and DWI sequence. There is no statistical heterogeneity between different studies (P = 0.33), and fixed effect model can be still used for meta-analysis (I^2^ = 14%). The summary diagnostic OR was 0.72 (95% CI: 0.43–1.19). Funnel plot (Fig 3) shows concentrated distribution of each point with symmetrical funnel shape, which indicates that there is no publication bias (P = 0.305, Egger’s test).

### Meta-Analysis

**Fig 4:**
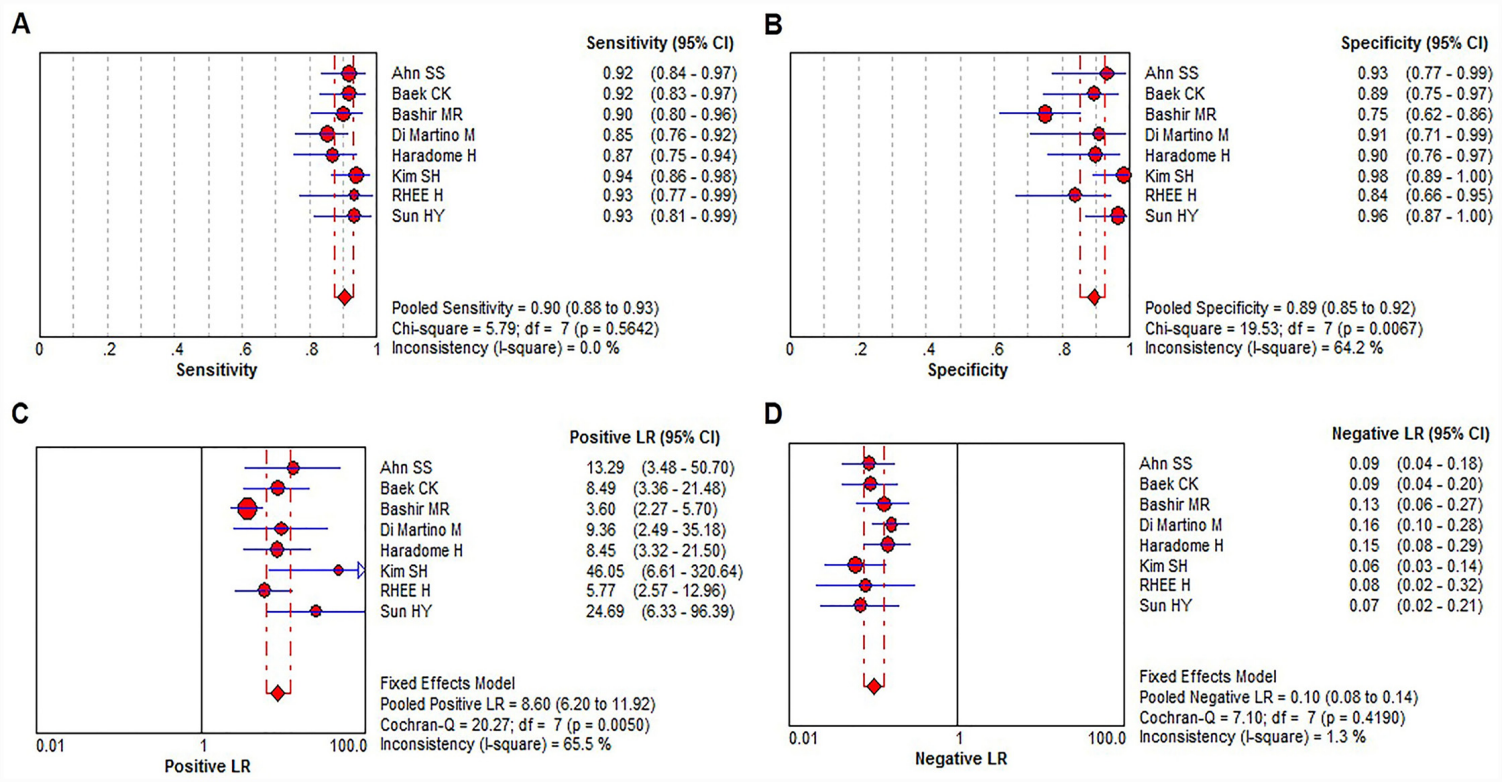
Forest plots of the estimates for Gd-EOB-DTPA-enhanced MRI in HCC diagnosis. (A) sensitivity analysis. (B) specificity analysis. (C) positive likelihood ratio analysis. (D) negative likelihood ratio analysis.

**Fig 5:**
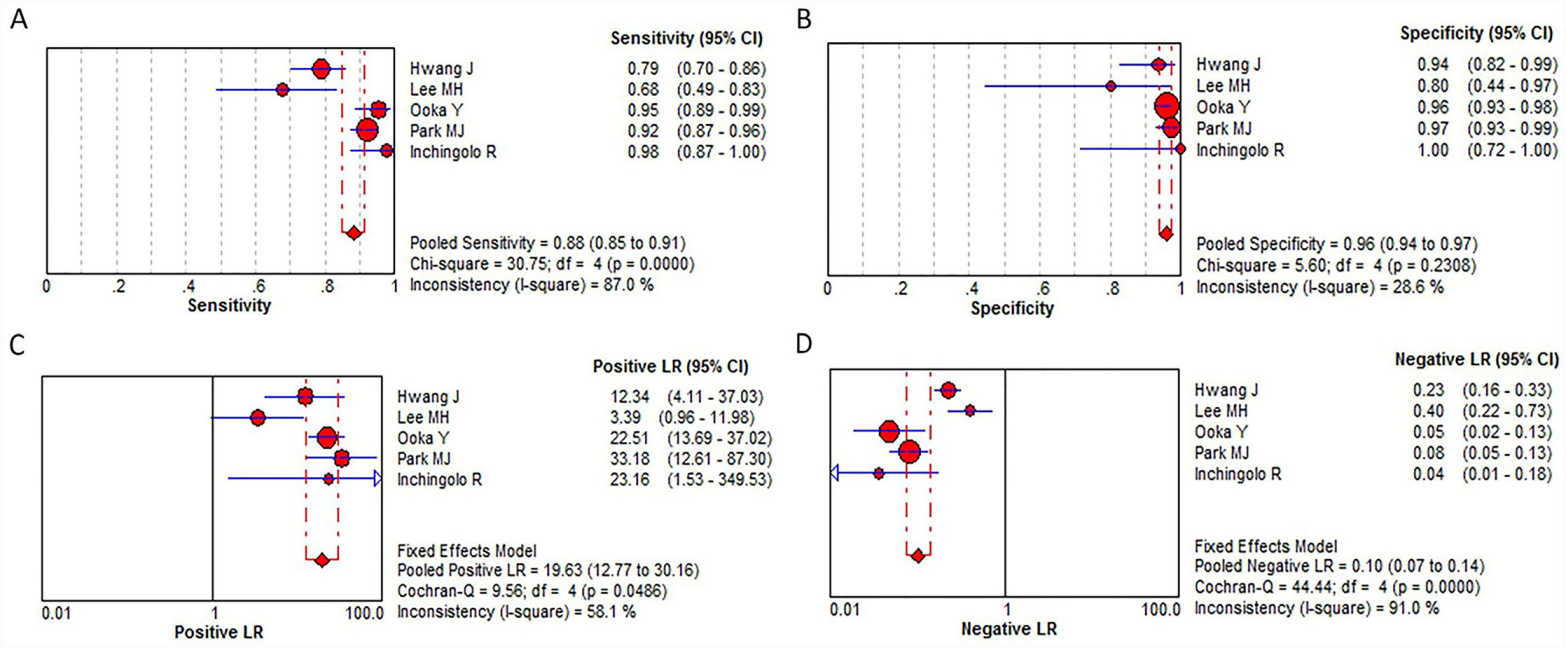
Forest plots of the estimates for combination of Gd-EOB-DTPA-enhanced MRI and DWI sequence in HCC diagnosis.

**Fig 6:**
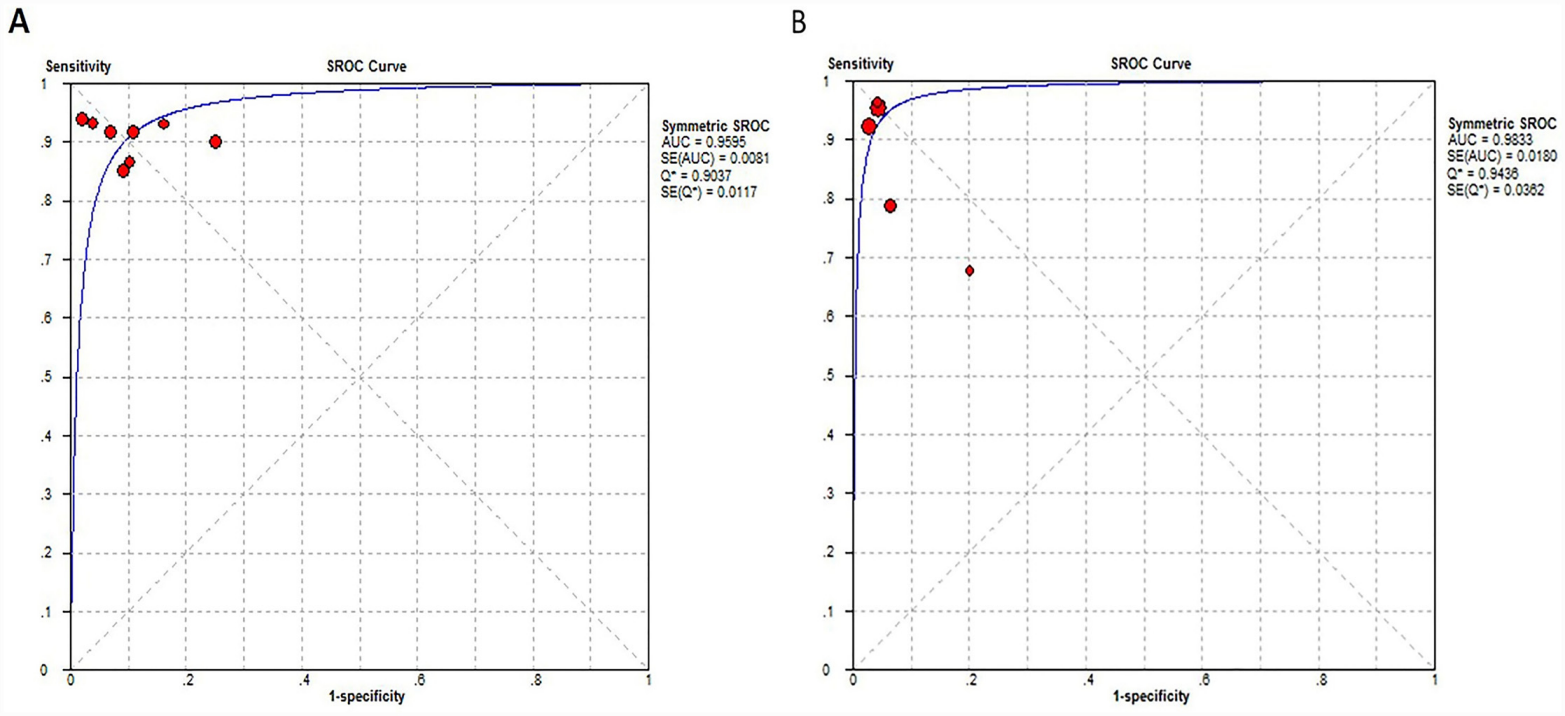
Summary receiver operating characteristic curves (SROC). (A) SROC curve for Gd-EOB-DTPA-enhanced MRI in HCC diagnosis. (B) SROC curve for combination of Gd-EOB-DTPA-enhanced MRI and DWI sequence in HCC diagnosis.

In 8 literatures regarding HCC diagnosis based on Gd-EOB-DTPA-enhanced MRI, the pooled sensitivity: 0.90 (95% confidence interval (CI): 0.88–0.93); specificity: 0.89 (95% CI: 0.85–0.92); positive likelihood ratio: 8.60 (95% CI: 6.20–11.92); negative likelihood ratio: 0.10 (95% CI: 0.08–0.14) were obtained (Fig 4). In 5 literatures relating to HCC diagnosis by combination of Gd-EOB-DTPA-enhanced MRI and DWI sequence, the pooled sensitivity: 0.88 (95% CI: 0.85–0.91), specificity: 0.96 (0.94–0.97), positive likelihood ratio: 19.63 (12.77–30.16), negative likelihood ratio: 0.10 (0.07–0.14) were obtained (Fig 5). As shown in Fig 6, in Gd-EOB-DTPA-enhanced MRI group and combined Gd-EOB-DTPA-enhanced MRI and DWI group, the area under the curve (AUC) of SROC were 0.9595 and 0.9833; Q* values were 0.9037 and 0.9436, respectively. The above data demonstrated that combined Gd-EOB-DTPA-enhanced MRI and DWI group had higher specificity, with no overlapping 95% confidence intervals, the positive likelihood ratio reached 19.63 with practical significance, and AUC value, the comprehensive evaluation index of diagnosis test, was higher than Gd-EOB-DTPA-enhanced MRI group (P = 0.019). The negative likelihood ratio and sensitivity were similar between two groups.

## Discussion

To the best of our knowledge, this is the first meta-analysis on the diagnostic performance of HCC using MR imaging with Gd-EOB-DTPA and DWI sequence. After systematic review and evaluation, this study included 13 literatures, among which 8 is to evaluate Gd-EOB-DTPA-enhance MRI, and 5 is to comprehensively evaluate Gd-EOB-DTPA-enhance MRI and DWI sequence in diagnosis of chronic liver disease associated HCC. In diagnostic method of combined evaluation, the pooled specificity was significantly higher than that in Gd-EOB-DTPA-enhance MRI, suggesting that the diagnostic methods greatly reduce misdiagnosis rate. In addition, the pooled positive likelihood ratio reaches 19.63, which indicates that the possibility of HCC is very high, if the diagnosis of the suspected cases is positive. However, one single index cannot reflect the whole test reaction, with only reference value. SROC curves, which are used to make a full and accurate evaluation on the diagnostic tests, are drawn according to the ratio of various studies. The results show that combined evaluation of Gd-EOB-DTPA-enhance MRI and DWI has higher AUC value than Gd-EOB-DTPA-enhance MRI alone (P<0.05). This indicates the combined diagnostic method can acquire more diagnostic information and make more accurate evaluation of HCC in chronic liver disease.

To investigate heterogeneity is the key to understand the possible factors affecting the estimation accuracy, and whether it is appropriate to evaluate combination of different studies. Threshold effect is one of the important causes for the heterogeneity of the diagnostic tests [39]. Threshold analysis was performed in studies on HCC diagnostic evaluation by combined diagnostic method and Gd-EOB-DTPA-enhance MRI alone, and the Spearman rank correlation coefficient were -0.429 (P = 0.289) and -0.700 (P = 0.188), respectively. This result indicates that the heterogeneity among sensitivity, specificity, positive likelihood ratio and negative likelihood ratio was not caused by the threshold effect. Subsequently, mate regression analysis was performed on literatures evaluating diagnostic value by Gd-EOB-DTPA-enhance MRI, to explore the sources of heterogeneity [39,40]. Evaluation covariates included case number, lesion number, lesion size, average age, the experiment design type and the equipment. The results showed that the above covariates were not sources of heterogeneity (P>0.05) (Table 4), which might be caused by limited number of the included literatures. Due to small number of included literatures in the comprehensive evaluation method, it is not suitable for mate regression analysis. Although the source of heterogeneity is not found, the combined statistics obtained from this study still have some reference value. On evaluation HCC diagnosis by Gd-EOB-DTPA-enhance MRI, the pooled sensitivity and specificity of our study are similar to those of Wu LM [41], with more precise 95% confidence interval. In the specificity analysis of the combined evaluation method, P value was 0.23 and I^2^ value was 28.6%, which indicates the results was homogeneous and have reference value.

**Table 4:** Meta regression analysis.

Variable	Coefficient	Standard error	P value	RDOR	[95%CI]
**Cte.**	32.418	14.3682	0.2656	—	—
**Mean age** ^a^	-0.495	0.2772	0.3252	0.61	(0.02;20.65)
**Patient**	-0.049	0.0358	0.4009	0.95	(0.60;1.50)
**Lession**	0.041	0.0505	0.5692	1.04	(0.55;1.98)
**Study design**	1.436	1.2792	0.4633	4.20	(0.00;48126481.50)
**Diameter** ^b^	-0.742	1.2436	0.6575	0.48	(0.00;3470717.50)
**Equipment**	-2.000	0.8555	0.8535	0.82	(0.00;43030.13)

^a^Baek CK [24] The average age was not recorded, and was replaced by the mean of the age range.

^b^Two categorical variable analysis was performed according to the diameter of liver cell carcinoma >3cm or ≤3cm.

RDOR, relative diagnostic odds ratios.

Combination of Gd-EOB-DTPA-enhanced MRI and DWI sequence has considerable significance in the diagnosis of HCC. Several studies reported that most HCCs with various degree of differentiation demonstrate hypointensity on gadoxetic-acidenhanced HBP images, which was due to the change in expression of OATP1B3, a liver-specific human drug transporter [3,42]. However, early-enhancing non-tumorous (EN) hepatic lesions may occasionally present with hypointensity during the hepatocyte phase, thus causing a diagnostic dilemma [43]. In addition, in clinical practice, HCCs frequently demonstrate atypical and inconclusive enhancement patterns different from the characteristic enhancement pattern of typical HCCs, and this may cause a delay in diagnosis [44]. Recently the Japan Society of Gastroenterology and Hepatology has proposed clinical practice guidelines and suggested that a nodular lesion showing an atypical imaging pattern on dynamic CT should be further examined by gadoxetic acid-enhanced MR imaging or contrast-enhanced ultra-sonography [45]. Inchingolo R et al. reported that suspicion should be raised for HCC, or at least high-grade dysplastic nodules (HGDN), when existing hyperintensity on DWI, especially in association with hypointensity on hepatobiliary phase, and low lesion-to-liver ratios, thus helping the characterization of atypically enhancing lesions [35]. Also, one study demonstrated that hyper-intensity on both T2WI and DW imaging are conducive to the diagnosis of hypervascular HCCs smaller than 1 cm [46]. Therefore, the addition of DW imaging to gadoxetic acid-enhanced MR imaging could be a promising strategy for both detection and characterization of HCC. Additionally, the authors of one recent research [33] suggest that a comprehensive evaluation using gadoxetic acid-enhanced MRI including a gradient dual-echo sequence and DWI is superior to CTAP/CTHA for the pre-therapeutic detection of HCC, regardless of nodule size.

Compared with other meta-analysis of diagnostic tests, this study has the following advantages: (1) In comprehensive evaluation on HCC diagnosis by Gd-EOB-DTPA-enhance MRI and DWI, all the gold standard was pathological examination; (2) All included literatures are acquired after detailed and clear literature screening process, and all cases had a history of chronic liver disease; (3) Before meta-analysis, heterogeneity analysis was performed using Review Manager 5.3 software, and to ensure homogeneity between various included studies; (4) More detailed data extraction was performed in all literatures for in-depth analysis and research.

The limitations of this study are as follows: firstly, the number of included literatures is limited; especially there are only 5 literatures on comprehensive consideration method. Though the included literature was small, the total number of subjects was up to 945 cases, including 460 cases with comprehensive consideration method. Of the total 1385 lesions, 665 lesions were diagnosed by comprehensive consideration method. All literatures were screened by QUADAS tools, with high quality and representative for each study. Secondly, most included literatures were retrospective studies, with only 2 prospective studies. However, the final diagnosis was not known for all researchers in the process of imaging diagnosis. In addition, the gold standard used was pathological examination or imaging follow-up, which was in accordance with the clinical diagnostic criteria, and can make accurate judgments of HCC.

## Conclusion

Compared with Gd-EOB-DTPA-enhance MRI, combined evaluation method increases diagnosis accuracy and specificity of HCC in chronic liver disease. Further studies with larger sample remain to be needed to investigate diagnostic value in HCC by combined application of Gd-EOB-DTPA-enhance MRI and DWI.

## Supporting Information

S1 Fig PRISMA 2009 Flow Diagram. (DOC)

S1 Table PRISMA 2009 Checklist. (DOC)
