# Supplementary material for: Combined Application of Gadoxetic Acid Disodium-Enhanced Magnetic Resonance Imaging (MRI) and Diffusion-Weighted Imaging (DWI) in the Diagnosis of Chronic Liver Disease-Induced Hepatocellular Carcinoma: A Meta-Analysis
Source: PLoS One. 2015 Dec 2;10(12):e0144247. doi: 10.1371/journal.pone.0144247 (PMC4668097; doi:10.1371/journal.pone.0144247)
Supplement: S1 Fig — (DOC) [file pone.0144247.s001.doc]

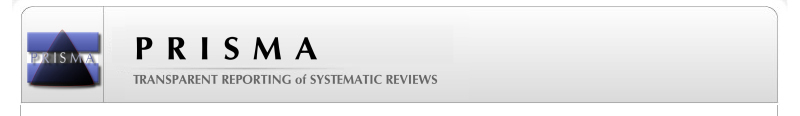
**PRISMA 2009 Flow Diagram**

**Screening**

**Included**

**Eligibility**

**Identification**

Records identified through database searching
(n = 1467 )

Additional records identified through other sources
(n = 0 )

Records after duplicates removed
(n = 848 )

Records screened
(n =34 )

Records excluded
(n = 814 )

Full-text articles assessed for eligibility
(n =16 )

Full-text articles excluded, with reasons
(n =18 )

Studies included in qualitative synthesis
(n =16 )

Studies included in quantitative synthesis (meta-analysis)
(n = 13 )
